# Evaluating feed value of native Jeju bamboo (*Sasa quelpaertensis* Nakai) for beef cattle

**DOI:** 10.5713/ab.22.0160

**Published:** 2022-09-02

**Authors:** Seul Lee, Youl Chang Baek, Mingyung Lee, Seoyoung Jeon, Han Tae Bang, Seongwon Seo

**Affiliations:** 1 Animal Nutrition and Physiology Division, National Institute of Animal Science, Rural Develpoment Administration, Wanju 55365, Korea; 2 Division of Animal and Dairy Sciences, Chungnam National University, Daejeon 34134, Korea

**Keywords:** *Bos Taurus coreanae*, Feed Evaluation, Hanwoo, Roughage, *Sasa quelpaertensis*

## Abstract

**Objective:**

Recently, indigenous Korean grass *Sasa quelpaertensis* Nakai (SQ) has garnered much interest as a roughage source for livestock to mitigate its adverse effects on habitat diversity. Thus, the objective of the present study was to evaluate the ruminal fermentation, palatability, and nutrient digestibility of SQ for Korean native beef cattle (Hanwoo) using *in vitro* rumen fermentation, *in situ* rumen degradability, and *in vivo* feeding trials.

**Methods:**

Using *in vitro* tests with rumen fluid as the inoculum for 48 h, ruminal fermentation of SQ was evaluated and compared with that of other roughage sources commonly used in Korea (i.e., rice straw, Timothy hay, and Italian ryegrass [IRG]). Additionally, an *in situ* trial 96 h was performed using three cannulated Hanwoo steers. Further, an *in vivo* trial was performed using eight Hanwoo steers to compare the palatability of SQ with rice straw in total mixed ration (TMR) and forage-concentrate separate feeding conditions. Finally, an *in vivo* digestibility trial of SQ fed as TMR of two particle sizes was performed with four Hanwoo steers.

**Results:**

*In vitro* and *in situ* trials revealed that SQ was comparable or superior to rice straw in terms of the ruminal fermentation characteristics of pH, gas production, total volatile fatty acid content, and effective ruminal dry matter digestibility (DMD), although its fermentability was lower than that of Timothy hay and IRG. In the palatability test, steers showed a greater preference for SQ when given as TMR. The total tract DMD of SQ fed as TMR was 75.9%±1.37%, and it did not differ by particle size.

**Conclusion:**

The feed value of SQ as a roughage source for Hanwoo steers is comparable or superior to that of rice straw, particularly when provided as TMR.

## INTRODUCTION

*Sasa quelpaertensis* Nakai (SQ), commonly called Jeju-Joritdae, is an indigenous broad-leaved bamboo grass that grows on Mount Halla, Jeju Island, South Korea. The genus *Sasa* is widespread in Asian countries, including Korea, Japan, China, and Russia [1]. In the Mount Halla National Park, SQ covers approximately 76% of the northern slopes, and the spread of SQ has damaged the mountain’s plant ecosystem diversity through its effects on light, temperature, humidity, and soil. SQ is an indeciduous perennial plant, and has a rhizome developing within the soil. SQ starts to occur in April, yields the highest in August, and the edge of the leaves begins to fade around September [2]. Therefore, SQ must be controlled for the ecological preservation of Mount Halla by directing its use as a resource for other purposes. Although many studies have explored the health-related functions, such as antidepressant-like, anti-cancer, and anti-inflammatory effects, of SQ components, other strategies capable of utilizing SQ at large amounts are required [3,4]. In this context, the use of SQ as a roughage source for herbivorous livestock is currently of great interest.

Several studies have explored the effects of grazing her bivores on Sasa species to maintain vegetation diversity. *S. nipponica* showed the highest biomass, growth rate, and crude protein (CP) content in summer, and it could thus be used as a forage source for deer [5]. Furthermore, *S. nipponica* could be supplied as feed to grazing cattle and horses, and the grazing behavior of these animals expanded with increasing feeding scale, with a similar foraging hierarchy [6]. In another study, the yield and growth characteristics of SQ produced by horse grazing were investigated, and the nutritional values and digestibility of the current-year-sprouted SQ tended to be higher than those of the previous-year-sprouted one [7]. Moreover, SQ could be used as a substitute to orchard grass when supplied as a total mixed ration (TMR) feed to horses [8]. Therefore, it is possible to use SQ as a forage source for ruminants. However, case studies on actual feeding are lacking.

To this end, the objective of the present study was to investi gate the feed value of SQ as a forage source for cattle. The nutritional value of feed can be analyzed using three methods: chemical, biological, and nutritional modelling. In the present study, we used chemical and biological methods to assess the nutritive value of SQ. Specifically, chemical methods included assessments based on the nutrients and fibers of feedstuff. Meanwhile, biological evaluation was based on the assessment of nutritional availability of feed using *in vivo*, *in situ*, and *in vitro* experiments. These methods focused on the digestibility and absorption of feed nutrients available for the metabolism of ruminants. Moreover, the effects of chopping time and particle size on the *in vivo* apparent nutrient digestibility of SQ were simultaneously evaluated. In addition, a comparative analysis of ruminal fermentation and degradability between SQ and other roughages commonly used in Korea (i.e., rice straw, Timothy hay, and Italian ryegrass [IRG]) was conducted. Finally, the palatability and total tract digestibility of SQ were evaluated and compared with those of rice straw.

## MATERIALS AND METHODS

### Ethics statement

All animal experimental procedures were reviewed and approved by the Institutional Animal Care and Use Committee of the National Institute of Animal Science (No. 2018-282).

### Sample preparation and animals for *in vitro* and *in situ* experiments

SQ hay, rice straw, Timothy hay, and IRG were used for *in vitro* and *in situ* rumen fermentation studies. 700 kg of newly sprouted SQ was manually harvested from a 2 ha organic harvesting site of the Jeju Resources Plant Research Center at Mount Halla. The grass was harvested during early July when SQ growth is at the peak. Upon harvest, the SQ samples were screened to remove other species and then transported to the laboratory. For nutrient analysis, SQ was chopped with a straw cutter in ~15-cm-long pieces, oven-dried at 60°C for 72 h, and then ground in a cyclone mill (Foss, Hillerød, Denmark). For chemical analysis (Table 1) and *in vitro* study, SQ was passed through 1 and 2 mm screens, respectively. The other three types of roughage were purchased from a domestic distributor and similarly treated for further analyses.

Three cannulated Hanwoo steers ( *Bos Taurus coreanae*, 30-month old, 542±22.5 kg) were included as donors. The donor animals were fed twice a day with 8 kg concentrate and 2 kg rice straw as fed basis. Water and mineral blocks (Rincal-block, Daehan Nupharm, Hwaseong, Korea; 50,000 IU vitamin (vit) A, 50,000 IU vit D_3_, 150 g Ca, 100 g P, 5,000 mg Fe, 2,500 mg Zn, 2,000 mg Mn, 2,000 mg Mg, 30 mg Na, 300 mg K, 6 mg Co, 6 mg I, and 380 g NaCl per 1 kg) were provided *ad libitum*.

### *In vitro* fermentation experiment (Exp 1)

The roughage sources used for *in vitro* fermentation were SQ hay, rice straw, Timothy hay, and IRG. Rumen liquid was collected 1 h before morning feeding and squeezed through four layers of cheesecloth before pH measurement. Rumen fluid from the three donors was pooled and combined with McDougall’s buffer at a 1:4 ratio under strictly anaerobic conditions [9], followed by the addition of 50 mL of inoculum (n = 3 per treatment). The control setup included three blank flasks containing only inoculum. Each treatment and control flask contained 0.5 g of fermentation substrates. The flasks were sealed with butyl rubber stoppers and aluminum caps and then incubated for 48 h at 39°C.

After 48 h of incubation, rumen fluid pH and total gas production were measured using a glass syringe (100 mL; Truth, Pvt. Ltd., Rajasthan, India). Rumen fluid was centrifuged at 6,000×*g* for 15 min at 20°C. The supernatant was used for volatile fatty acid (VFA) analysis. For VFA analysis, 25% metaphosphoric acid solution (Catalog number 239275; Sigma-Aldrich, St. Louis, MO, USA) was added to the rumen fluid at 10% of total volume. VFA concentration was determined as described by Erwin et al [10]. The supernatant was injected into a gas chromatograph (6890N; Agilent Technologies, Santa Clara, CA, USA) equipped with a flame ionization detector and capillary column (Nukol Fused silica capillary column 15 m×0.53 mm×0.5 μm; Supelco Inc., Bellefonte, PA, USA). The oven, injector, and detector temperatures were 110°C, 250°C, and 250°C, respectively. Nitrogen was used as the carrier gas at a flow rate of 25.0 mL/min. Ammonia concentration was analyzed using the method described by Chaney and Marbach [11]. The samples were stored in a deep freezer (−80°C) until analysis. The samples were centrifuged (6,000×*g* for 15 min at 4°C) to obtain 20 μL of supernatant. The supernatant, distilled water, and ammonia standard solutions (2.5, 5, 10, 20, and 40 mg NH_3_-N/100 mL) were added to three sets of 20 mL tubes. Then, 1 mL of phenol color reagent (distilled water 1 L; phenol [C_6_H_5_OH] 50 g; sodium nitroferricyanide [Na_2_(Fe[CN]_5_NO)·2H_2_O] and alkali-hypochlorite reagent [distilled water 1 L, sodium hydroxide (NaOH) 25 g, and sodium hydrochloride (4% to 6% NaOCl)] 16.8 mL) were added to each tube and mixed. The tubers sealed with a butyl rubber stopper and incubated at 37°C in a constant-temperature water bath for 15 min. After incubation, 8 mL of distilled water was added to measure absorbance at 630 nm using a microplate reader (Molecular Devices, San Jose, CA, USA). Ammonia concentration was determined using a standard calibration curve. All analyses were repeated three times, and the mean values were used.

### *In situ* degradation experiment (Exp 2)

An *in situ* ruminal degradation experiment was performed using three cannulated Hanwoo steers. Test diet samples, SQ hay, rice straw, Timothy hay, and IRG were dried and passed through a 2 mm mesh screen in a cyclone mill (Foss, Denmark) before rumen incubation. Nylon bags (NL 130-030/330PW, NBC Inc., Tokyo, Japan), each approximately 8×15 cm (45 μm pore size; surface area = 41.67 mg/cm^2^), were filled with 5 g of dried, ground feed and tied with a rubber band.

Nylon bags (four samples × six incubation times × three replicates) were inserted in the ventral rumen of each animal. The bags were removed at the end of each incubation period (6, 12, 24, 48, 72, and 96 h), washed in a washing machine for 30 min, and dried to a constant weight in a 60°C forced air oven for 48 h. Then, the residual dry matter (DM) content was determined.

*In situ* rumen DM degradability was estimated by applying time-series data to the model described by Øskov and McDonald as follows [12]:


(1) $$\text{P}=\text{a}+\text{b }(1-{exp}^{-k_{d}.dt})$$

where P is rumen degradation (%), a is the soluble fraction (%), b is the slowly degradable (potentially degradable) fraction (%), k_d_ is the fractional rate of disappearance (h^−1^), and t is the time of incubation (h).

The effective degradability (ED) of nutrients, which is an estimate of the ruminal degradability of nutrients accounting for the continuous flow of digesta in the rumen [12], was calculated using the following equation:


(2) $$\text{ED }(\%)=\text{a}+\text{b}\times \frac{k_{d}}{k_{d}+k_{p}}$$

where ED is effective degradability; a, b, and k_d_ are constants of the rumen-degradation model; and kp is the fractional rate of passage out of the rumen, assumed to be 0.02, 0.04, and 0.06 h^−1^ [13].

### Sample preparation and for *in vivo* experiments

For *in vivo* trials, SQ and rice straw were prepared as silages instead of hays, because rice straw is commonly provided to cattle as silage in the field. The overall silage preparation procedure was the same for SQ and rice straw. Briefly, the roughages were sprayed with a silage fermentation solution (1 L/t) prepared by mixing the silage fermentation reagent (Silac S, GoodBio, Wanju, Korea) containing *Lactobacillus plantarum*, *L. acidophilus*, and *Bacillus subtilis* with tap water (1:1 dilution). The inoculated roughages were then wrapped with two layers of vinyl and one layer of feed bag under vacuum conditions. The silages, packed in 35 kg bags, were used for subsequent *in vivo* digestibility and palatability trials after a month of anaerobic fermentation.

### *In vivo* palatability test (Exp 3)

A total of eight Hanwoo steers (32-month old, 439±76.34 kg) participated in this 12-day palatability trial. Each steer was individually housed in a separate pen. The animals were fed twice daily (09:00 and 16:00); water and mineral blocks were provided *ad libitum*.

Palatability was assessed using the preference test described by Kawamoto et al [14] and Ali et al [15], which measures the preference for a feed based on its intake, with modified feeding times. Voluntary feed intake per body weight (BW) was used as the criterion for feed palatability. The acceptance of SQ silage was assessed as the relative amount of SQ silage consumed over rice straw silage under both TMR and roughage-concentrate separate feeding conditions. The difference in roughage intake, representing the relative preference of SQ silage to rice silage, was determined by subtracting rice straw intake from SQ intake on a DM basis.

The experimental period was divided into four periods of three days each. The experimental unit was an individual steer. The steers were rotationally provided the diet by one of the two feeding methods (i.e., roughage-concentrate separate feeding and TMR feeding) in each period. The treatment was the feeding method, which was duplicated with eight replications. With this experimental arrangement, effect of the feeding methods on comparative consumption of SQ silage and rice silage could be evaluated although the feeding method was partily confounded with the period. The same roughages and concentrate mix were used throughout the experimental period. In the first and third periods, rice straw silage, SQ silage, and concentrate mix were provided separately. The formula of the concentrate mix and chemical composition of the feed are summarized in Tables 2 and 3. Rice straw silage and SQ silage were fed simultaneously to offer steers the choice to consume the preferred roughage. Both silages were provided for 40 min and then removed to measure the remainder. The concentrate mix was then provided for another 40 min and removed to measure intake. The experimental diets were offered as TMR in the second and fourth periods. The forage-to-concentrate ratio of TMR was set to 15:85 on a DM basis, being the same as that set in the *in vivo* digestibility trial. Two types of TMR containing either rice straw silage or SQ silage were provided simultaneously, and the steers were offered free choice to consume any of the two TMRs. After offering the TMR for 40 min, leftovers were measured to estimate the voluntary intake of individual steers.

### *In vivo* digestibility trial (Exp 4)

Four Hanwoo steers (29-month old, 474±28.15 kg) were subjected to an *in vivo* digestibility trial. The animals were housed individually in metabolic cages (127×250×200 cm width× depth×height) to collect orts and feces for measuring the apparent digestibility of the total tract.

The experiment followed a 2×2 crossover design with two periods and lasted 36 days. The treatment was the mixing time of preparing SQ TMR (either 7 or 40 min), and the steers were randomly assigned to two replicates of two cows each. For replicate one, during the first period of the treatment, they were given SQ TMR of 7 min mixing time; and during the second period, they were given SQ TMR of 40 min mixing time. The second replicate was treated reversely (first period, SQ TMR of 40 min mixing time; second period, SQ TMR of 7 min mixing time). Since TMR was also chopped with blades during mixing, the particle size of the TMR decreased as the mixing time increased. Each period lasted 18 days, including 14 days for feed and metabolic cage adaptation and 4 days for fecal collection.

The formula of the experimental diets is summarized in Table 2, and the chemical composition of the feed is presented in Table 4. The forage-to-concentrate ratio of TMR was 15:85 on a DM basis. Particle size distribution was measured using a two-screen (19 and 8 mm) Penn State Particle Separator [16] and expressed as the percentage of each fraction in weight on a DM basis. According to the Korean Feeding Standard for Hanwoo [17], animals were fed to the maintenance level (1.23% of initial BW [DM basis]), and roughage was supplied at <15% of dietary DM, as recommended for the late fattening stage. Steers were fed twice at 09:00 and 16:00, and drinking water was provided *ad libitum*.

### Nutrient analysis

All analyses were performed in triplicate. DM (#930.15), CP (#990.03), acid detergent fiber (ADF; #973.18), ash (#942.05), and ether extract (EE; #2003.05) in the feed and fecal samples were determined using the corresponding AOAC methods [18]. Neutral detergent fiber was analyzed using heat-stable amylase and expressed inclusive of residual ash (aNDF), as described previously [19]. Similarly, soluble protein (SOLP) [20], neutral detergent insoluble crude protein (NDICP), and acid detergent insoluble crude protein (ADICP) per sample were determined following previously described procedures [21]. Non-fiber carbohydrates (NFC) content was calculated as 100 − ash − EE − CP − (aNDF − NDICP) based on NRC guidelines [22]. Dietary protein fractions were estimated according to the Cornell Net Carbohydrate and Protein System, with previously described modifications [23].

### Statistical analysis

Statistical analyses were conducted according to the guidelines proposed by Seo et al [24]. Data in exp 1,2, and 4 were analyzed using the GLIMMIX procedure in SAS (v. 9.4, SAS Institute Inc., Cary, NC, USA). Data in exp 3 were analyzed using the MIXED procedure in SAS according to following linear model:


$$y_{ijk}=\mu +\tau_{i}+a_{j}+{ɛ}_{ijk}$$

Where *y*_*ijk*_ is *k*th observation (i.e., feed intake) in *i*th feeding method with *j*th animal, μ is the overall mean, τ_*i*_ is the fixed effect of *i*th feeding method (*j* = 1 to 2), *a*_*j*_ is the random effect of jth animal (*i* = 1 to 8), and *ɛ*_*ijk*_ is the unexplained random effect on kth observation in ith feeding method with *j*th animal. Data in Exp 4 were analyzed using the TTEST procedure in SAS. In Exp 1 and 2, to compare ruminal fermentation and degradability of SQ with that of rice straw, Timothy hay, and IRG, data were analyzed as a repeated observation (Exp 1, n = 3; Exp 2, n = 3) on each experimental unit (Exp 1, flask; Exp 2, nylon bag). In Exp 3 and 4, data were analyzed as a repeated observation (Exp 3, n = 16; Exp 4, n = 4) on each experimental unit (animal). Inter-treatment differences were compared using Tukey’s range test to determine the significance of the overall treatment effect [24]. Statistical significance was declared at p<0.05 and a trend was discussed at 0.05≤p<0.1.

## RESULTS

### *In vitro* fermentation (Exp 1)

SQ contained more CP and NDICP compared to other roughages (Table 1). As a result of protein fraction analysis, the PA+B1 fraction content of SQ was the lowest, and the PB2 and PB3 contents were the highest out of the four roughages. SQ and rice straw contained more aNDF than Timothy hay and IRG.

*In vitro* rumen fermentation characteristics differed among the four roughages (p<0.01). However, the result of SQ did not show a difference with rice straw in terms of rumen pH, gas production, and NH_3_-N (Table 5). Rumen pH was significantly higher in the SQ and rice straw treatments than in the Timothy hay and IRG treatments (p<0.01). NH_3_-N was not significantly different between roughage types, although gas production was significantly lower in the SQ and rice straw treatments than in the Timothy hay and IRG treatments (p<0.01). The total VFA content was significantly higher in the Timothy hay and IRG treatments (p<0.01). Acetate, propionate, and valerate contents were higher in the IRG treatment, but butyrate content remained unchanged. Furthermore, the A:P ratio was higher in the SQ treatment than in the other treatments (p<0.01).

### *In situ* degradation (Exp 2)

The *in situ* DM degradability was significantly lower in the SQ (53.0%) and rice straw (53.7%) treatments than in the Timothy hay (65.1%) and IRG (63.4%) treatments after 96 h of rumen degradation (Table 6, p<0.01). The rumen degradability of NDF and ADF was the highest in the Timothy hay treatment, followed by IRG, rice straw, and SQ treatments (p<0.01). The rapidly degradable a fraction was significantly higher in the SQ treatment (36.5%; p<0.01) than in the other treatments (Table 7). The fractional degradation rate of insoluble but degradable b fraction was the lowest in the SQ treatment (0.009 h^−1^), and the proportion of b fraction tended to decrease in the other treatments (p = 0.07). The potential degradability rate (a+b fraction) was higher in the Timothy hay treatment (72.2%), but lower in the IRG (68.1%), SQ (65.2%), and rice straw (58.1%) treatments, although these differences were not significant. Effective ruminal DM degradability in the SQ treatment was comparable to that in the Timothy hay and IRG treatments and higher than that in the rice straw treatment (p<0.01). As rumen outflow increased, ED_DM_ decreased steadily, ranging from 44.7% to 39.9% in the SQ treatment. At all measurement rates, ED_DM_ was the lowest in the rice straw treatment (p<0.01).

### *In vivo* palatability and nutrient digestibility (Exp 3 and 4)

Feeding methods (i.e., separate vs TMR feeding) altered the forage and concentrate intake while maintaining the same total dry matter intake (DMI) (Table 8). Total daily DMI was 1.87% BW on average, which did not differ between the feeding methods. However, concentrate DMI was 0.18% BW higher in separate feeding than in TMR feeding (p< 0.001). Moreover, total forage consumption was higher in TMR feeding than in separate feeding (0.12% BW vs 0.28% BW; p<0.001). Interestingly, the increase in forage intake during TMR feeding was due to the increased consumption of SQ. The intake of rice straw did not differ between the two feeding methods (0.1% BW); however, the daily consumption of SQ was significantly higher when it was supplied as TMR (p<0.001). In separate feeding, steers consumed little SQ. The daily consumption of SQ was significantly lower than that of rice straw in separate feeding, but it was significantly higher than that of rice straw in TMR feeding (p<0.001). The difference in roughage intake was significantly higher in TMR than in separate feeding (p<0.001).

Although mixing time affected the particle size of SQ TMR, it did not affect its DM digestibility (7 min, 75.6%; 40 min, 76.1%) (Table 9). When mixed for 7 min, the distribution of large particles (>19 mm) tended to differ according to the mixing time (7 min, 2.2%; 40 min, 0.5%; and p = 0.06). Moreover, mixing time affected the distribution of small particles (0 to 8 mm; 7 min, 64.8%; 40 min, 75.0%; p = 0.02) and medium particles (8 to 19 mm; 7 min, 33.0%; 40 min, 24.5%; p<0.05). Mixing time did not significantly affect CP (7 min, 79.1%; 40 min, 76.1%), NDF (7 min, 49.4%; 40 min, 49.9%), and ADF digestibility (7 min, 34.3%; 40 min, 29.2%). The four animals showed an average initial weight of 474.3±28.2 kg and an average end weight of 468.4±23.1 kg.

## DISCUSSION

This study was designed to evaluate the nutritional value of SQ as an alternative to conventional roughage sources. Although several studies have been conducted on the utilization of SQ, studies on its use as a roughage source for cattle are scarce. In this light, the present study successfully evaluated the feed value of SQ as a substitute roughage source, particularly rice straw, for beef cattle.

Nutrient composition and *in vitro* rumen fermentation analyses revealed that the nutritional value of SQ was comparable to that of rice straw but lower than that of Timothy straw and IRG. These findings are consistent with the results of previous *in vitro* fermentation studies on domestic roughage, including IRG and rice straw [25]. After 48 h of incubation, IRG and rice straw showed a higher pH (p<0.01), lower gas production, and lower total VFA concentration (p<0.01). The NFC content of SQ, rice straw, Timothy hay, and IRG was respectively 15.2%, 10.8%, 29.3%, and 23.7%. NFCs in feedstuffs serve as a source of fermentable carbohydrates, such as starch, sugar, and pectin. Moreover, NFC content is positively correlated with the feed energy content, degree of microbial protein synthesis in the rumen [22], gas production on a DM basis, and total VFA (specifically propionate and butyrate) content [26]. In the present study, the NFC and total VFA contents following ruminal fermentation were higher in Timothy hay and IRG.

*In situ* degradability results confirmed that Timothy hay is a relatively high-quality feedstuff with a high degradation rate, potential degradability, and ED. In contrast, rice straw is considered to be of inferior quality; indeed, we noted that the DM degradation rate, potential degradability, and ED of rice straw were the lowest. The result of high ruminal degradability of Timothy hay compared to rice straw is consistent with findings of a previous study on the *in situ* degradability of various roughages [27], in which Timothy hay was shown to present a superior DM and CP ruminal disappearance rate to rice straw. In the present study, the fractional degradation rate of the insoluble but degradable b fraction in SQ was the lowest at 0.009 h^−1^, suggesting that the b fraction of SQ was hardly degraded. However, the effective ruminal DM degradability of SQ was comparable to that of Timothy hay and IRG and superior to that of rice straw due to the large a fraction. This outcome is attributed to the physical composition of SQ, which comprises a solid stem and large, soft leaves.

In *in vivo* trials, SQ were made into silage to extend the storage period. In Korea, roughage is mainly processed into hay or silage, and the frequency of silage production is higher in the field because of its advantages. For instance, silage production is more feasible than hay production mainly because of frequent rains in Korea, and silage is more advantageous in terms of the ease of transport and storage [28]. TMR feeding allows for supplying a nutritionally balanced diet to all cows, rendering every bite of feed essentially complete [29]. In addition, TMR feeding is prioritized over separate feeding of concentrate mix and forage to increase nutrient use efficiency and rumen stability [30]. Prolonged mixing times reduce the particle size of TMR and enhance the uniformity of mixing. Thus, the effect of mixing time must be explored to standardize the methodology of using SQ in the field.

Several studies have been conducted on the effect of TMR on increasing feed intake in cattle. Gordon et al reported that TMR feeding increased animal feed intake due to the effect of mixing roughage and concentrate feed in their review of 13 studies [30]. On the contrary, some studies reported contradictory results regarding TMR and feed intake. Lee et al [31] reported that separate feeding of concentrate mix and forage did not significantly affect feed intake during the fattening period, whereas TMR feeding increased feed consumption during the growing period. Cattle at the fattening stage (32-month old) used in the current study, and their daily feed intake was not affected by the feeding method. This discrepancy can be attributed to differences in the composition of feed across countries, and in the nutrient according to the growth stage of cattle. For instance, the proportion of concentrate mix in the total feed is higher than that of forage, whereas in studies reviewed by Gordon et al [30], the proportion of forage was higher than that of the concentrate mix.

Though TMR feeding did not affect total feed intake, it significantly increased forage intake, especially for SQ. Although animals mostly consumed rice straw silage under separate feeding, they preferred SQ to rice straw silage when fed as TMR. Therefore, the palatability of SQ relative to rice straw improved with TMR feeding rather than with separate feeding. This improvement in the palatability of SQ TMR is considered to result from to the effect of the feeding method. Another factor affecting the palatability of SQ silage is particle size. In particular, the decrease in particle size under TMR feeding compared with that under separate feeding improved the palatability of SQ, as palatability is associated with the physical properties of the feed. In a previous study on correlations between feed properties and feed intake of dairy cows, DMI and NDF intake increased with decreasing particle size [32]. In another study on the association between roughage length and DMI of small ruminants (sheep and goats), intake was higher when roughage was shorter [33]. Although SQ and rice straw TMR were subjected to the same physical treatment, the preference for the former was likely altered significantly. Taken together, the feed value of SQ is comparable or superior to that of rice straw, particularly when supplied in a more palatable form, such as TMR.

The reduction in particle size due to the prolongation of mixing time did not alter the *in vivo* total tract apparent nutrient digestibility of SQ TMR. Particle size plays an important role in digestibility [34,35]. The digestibility of DM, ADF, and protein tends to decrease with increasing particle size of feed, which increases the intake of physically effective NDF, resulting in a longer chewing time [36]. However, our *in vivo* results indicated that the difference in mixing and cutting times did not affect the digestibility of SQ TMR, regardless of the altered the feed particle size. This similar DM digestibility likely resulted from the insufficiency of roughage length to affect the physical effectiveness of NDF. Another reason is that the SQ accounted for only 15% of the total feed due to the relatively high proportion of concentrate mix on a DM basis. Given the lack of correlation between mixing time and feed digestibility, farmers may not require to extend the TMR mixing time to increase the digestibility of SQ.

The ultimate goal of the present study was to contribute to the conservation of the plant ecosystem diversity on Mount Halla by promoting the utilization of SQ as a feed resource. Our experiments revealed that the rumen degradability of SQ is similar to that of rice straw. Rice straw is largely used as a low-nutrition feed by farmers in Korea, and SQ may serve a similar purpose in Hanwoo steers, possibly offering even a slightly greater feed value than rice straw. Nevertheless, further *in vivo* experiments are warranted before SQ is applied in the field. In addition, the feasibility of substituting rice straw with SQ, given their similar fermentation and degradation properties, must be explored. Reducing the particle size of SQ by extending the mixing duration did not improve its *in vivo* digestibility; nonetheless, when offered both SQ and rice straw in the form of TMR, steers preferred the former. However, when roughage and concentrate were offered separately, the animals preferred rice straw over SQ as the roughage source. In conclusion, SQ can be used as cattle feed to mitigate its negative environmental impact, and it should ideally be provided in the form of TMR.

## Figures and Tables

**Table 1:** Chemical composition of feeds used in *in vitro* and *in situ* experiments (g/kg DM, unless otherwise stated)

Item	SQ	Rice straw	Timothy hay	Italian ryegrass
DM (g/kg as fed)	675	676	955	742
CP	87	63	69	78
SOLP	9	18	25	34
NDICP	30	21	7	10
ADICP	20	19	6	7
NFC	152	108	293	237
aNDF	673	711	585	632
ADF	501	523	367	420
Ash	118	141	60	63
Ca	3	5	2	1
P	0.6	1.3	2	1
Mg	0.5	1.8	1.1	1.3
K	4.7	22.7	19.0	17.8
Na	0.4	0.7	0.3	3.9
Fe (ppm)	92	1,428	65	381
Mn (ppm)	497	1,469	48	100
Zn (ppm)	21	43	31	19
Protein fractions^1)^ (g/kg CP)				
PA+B1	103	286	362	436
PB2	556	378	532	437
PB3	113	33	26	40
PC	228	303	80	87

SQ, *Sasa quelpaertensis* Nakai; DM, dry matter; CP, crude protein; SOLP, soluble CP; NDICP, neutral detergent insoluble CP; ADICP, acid detergent insoluble CP; NFC, non-fiber carbohydrates; aNDF, neutral detergent fiber analyzed using heat-stable amylase and expressed inclusive of residual ash; ADF, acid detergent fiber.

1) PA: protein A fraction, PA (%CP) = non-protein nitrogen (NPN) (%SOLP)×0.01×SOLP (%CP); PB1: protein B1 fraction, PB1 (%CP) = SOLP (%CP) − A (%CP); PB2: protein B2 fraction, PB2 (%CP) = 100 − A (%CP) − B1 (%CP) − B3 (%CP) − C (%CP); PB3: protein B3 fraction, PB3 (%CP) = NDICP (%CP) − ADICP (%CP); PC: protein C fraction, PC (%CP) = ADICP (%CP).

**Table 2:** Ingredients of the concentrate mix used in *in vivo* experiments (exp 3 and 4, % as fed)

Ingredient	Composition
Corn	40.8
Corn gluten feed	20.8
Wheat bran	13.4
Soybean meal	9.9
Coconut meal	4.1
Rapeseed meal	4.1
Palm meal	4.1
Limestone	1.8
Salt	0.5
Sodium bicarbonate	0.5

**Table 3:** Chemical composition of feeds used in the palatability test (g/kg DM, unless otherwise stated)

Composition	SQ silage	Rice straw silage	Concentrate mix
DM	396	344	871
CP	63	47	165
aNDFom	768	598	245
ADF	515	417	101
Ash	66	106	59
GE (Kcal/g)	4.6	4.1	4.4

SQ, *Sasa quelpaertensis* Nakai; DM, dry matter; CP, crude protein; aNDFom, NDF assayed with heat-stable amylase and expressed exclusive of residual ash; ADF, acid detergent fiber; GE, gross energy.

**Table 4:** Chemical composition of *Sasa quelpaertensis* Nakai TMR feed at different mixing times (g/kg dry matter, unless otherwise stated)

Composition	Treatment	

	SQ TMR (7 min)	SQ TMR (40 min)
DM	789	785
CP	148	156
EE	30	30
aNDFom	270	261
ADF	131	112
Ash	68	63
GE (Kcal/g)	4.4	4.4

SQ, *Sasa quelpaertensis* Nakai; TMR, total mixed ration; DM, dry matter; CP, crude protein; EE, ether extract; aNDFom, NDF assayed with heat-stable amylase and expressed exclusive of residual ash; ADF, acid detergent fiber; GE, gross energy.

**Table 5:** *In vitro* rumen fermentation characteristics and gas production at 48 h of incubation

Variable	Treatment				SEM	p-value

	SQ	Rice straw	Timothy hay	IRG		
pH	6.67^a^	6.65^a^	6.58^b^	6.58^b^	0.016	<0.01
NH_3_-N (mg/dL)	22.5	22.7	23.0	22.2	5.67	0.62
Gas production (mL)	89^b^	98^b^	108^a^	115^a^	2.8	<0.01
Total VFA (mM)	87.1^c^	93.4^b^	102.2^a^	103.0^a^	1.38	<0.01
Acetate (mM)	55.8^c^	58.5^bc^	62.5^ab^	64.7^a^	1.67	<0.01
Propionate (mM)	13.4^c^	16.8^b^	19.0^ab^	19.5^a^	0.83	<0.01
A:P ratio	4.2^a^	3.5^b^	3.3^b^	3.3^b^	0.07	<0.01
Butyrate (mM)	12.7	12.5	12.5	12.6	0.15	0.81
Valerate (mM)	1.4^c^	1.6^bc^	1.7^ab^	1.8^a^	0.06	<0.01

SQ, *Sasa quelpaertensis* Nakai; IRG, Italian ryegrass; SEM, standard error of mean; VFA, total volatile fatty acids; NH_3_-N, ammonia nitrogen; A:P ratio, acetate-to-propionate ratio.

a–c Values in the same column with different superscript letters are significantly different (p<0.05).

**Table 6:** *In situ* ruminal degradability of four roughage types (% dry matter basis)

Item	Time (h)	Treatment				SEM	p-value

		SQ	Rice straw	Timothy hay	IRG		
DMD	0	40.1^a^	26.2^b^	36.9^a^	36.3^a^	1.94	<0.01
	6	38.1^a^	21.4^c^	33.9^b^	21.4^c^	0.55	<0.01
	12	40.1^a^	26.2^b^	36.9^a^	36.3^a^	1.94	<0.01
	24	39.9^a^	32.9^b^	43.1^a^	40.8^a^	1.67	<0.01
	48	45.7^b^	43.9^b^	55.2^a^	53.5^a^	1.41	<0.01
	72	49.0^b^	50.9^b^	59.5^a^	59.0^a^	1.23	<0.01
	96	53.0^b^	53.7^b^	65.1^a^	63.4^a^	1.45	<0.01
NDFD	0	26.4^a^	11.1^c^	23.0^b^	6.9^d^	0.74	<0.01
	6	28.1^a^	13.8^c^	26.2^b^	14.2^c^	0.64	<0.01
	12	30.1^a^	17.3^b^	29.4^a^	20.8^b^	2.35	<0.01
	24	27.1^b^	25.2^b^	35.4^a^	26.2^b^	1.95	<0.01
	48	35.9^c^	39.1^c^	49.6^a^	43.7^b^	1.61	<0.01
	72	39.6^d^	46.9^c^	55.2^a^	51.3^b^	1.45	<0.01
	96	44.5^d^	49.9^c^	61.5^a^	56.8^b^	1.65	<0.01
ADFD	0	21.5^b^	9.7^c^	26.3^a^	7.7^c^	0.74	<0.01
	6	23.0^b^	9.0^d^	27.3^a^	11.0^c^	0.67	<0.01
	12	23.7^b^	12.6^c^	30.8^a^	19.2^b^	2.42	<0.01
	24	21.8^b^	20.1^b^	35.3^a^	24.1^b^	2.01	<0.01
	48	29.3^d^	35.2^c^	50.0^a^	41.7^b^	1.68	<0.01
	72	33.2^d^	43.3^c^	54.7^a^	49.9^b^	1.51	<0.01
	96	38.9^d^	46.5^c^	61.3^a^	55.5^b^	1.23	<0.01

SQ, *Sasa quelpaertensis* Nakai; IRG, Italian ryegrass; SEM, standard error of mean; DMD, dry matter degradability; NDFD, neutral detergent fiber degradability; ADFD, acid detergent fiber degradability.

a–d Values in the same column with different superscript letters are significantly different (p<0.05).

**Table 7:** *In situ* ruminal degradation characteristics of various roughage types (dry matter basis)

Item	Treatment				SEM	p-value

	SQ	Rice straw	Timothy hay	IRG		
a fraction^1)^	36.5^a^	16.5^d^	30.0^b^	25.3^c^	0.52	<0.01
b fraction^2)^	28.7	41.5	42.2	42.8	4.69	0.07
a+b Fraction^3)^	65.2	58.1	72.2	68.1	4.74	0.09
Kd^4)^	0.009^b^	0.023^a^	0.018^ab^	0.023^a^	0.0039	0.02
Effective degradability (ED_DM_)^5)^						
ED_DM2_	44.7^b^	38.5^c^	49.5^a^	47.3^ab^	0.82	<0.01
ED_DM4_	41.3^a^	31.5^b^	42.7^a^	40.2^a^	1.07	<0.01
ED_DM6_	39.9^a^	27.9^c^	39.5^ab^	36.6^b^	0.99	<0.01

SQ, *Sasa quelpaertensis* Nakai; IRG, Italian ryegrass; SEM, standard error of the mean.

1) a fraction: rapidly degradable a fraction (%).

2) b fraction: slowly degradable b fraction (%).

3) a+b fraction: potential degradability (%).

4) Kd: degradation rate of degradable b fraction (h^−1^).

5) The ED value was calculated using three passage rates: 0.02 h^−1^ (ED_DM2_), 0.04 h^−1^ (ED_DM4_), and 0.06 h^−1^ (ED_DM6_).

a–d Values in the same column with different superscript letters are significantly different (p<0.05).

**Table 8:** Daily feed intake of animals by feeding method (% BW, dry matter basis)

Item	Feeding method		SEM	p-value

	Separate feeding	TMR feeding		
Total intake	1.88	1.86	0.083	0.784
Concentrate mix	1.76	1.58	0.085	0.070
Forage	0.12	0.28	0.007	<0.001
SQ	0.01	0.18	0.015	<0.001
Rice straw	0.11	0.10	0.016	0.486
Roughage intake difference^1)^				
% BW	−0.09	0.06	0.029	<0.001

TMR, total mixed ratio; SEM, standard error of mean; SQ, *Sasa quelpaertensis* Nakai; BW, body weight.

1) Roughage intake difference (% BW, dry matter basis) = (SQ intake) − (Rice straw intake).

**Table 9:** Effect of mixing time on the particle size distribution and apparent *in vivo* nutrient digestibility of *Sasa quelpaertensis* Nakai TMR (% DM basis)

Item	Treatment		SEM	p-value

	SQ TMR (7 min)	SQ TMR (40 min)		
Particle size distribution (% dry matter basis)				
Small (0 to 8 mm)	64.8	75.0	2.27	0.02
Middle (8 to 19 mm)	33.0	24.5	1.92	<0.05
Large (>19 mm)	2.2	0.5	0.49	0.06
Apparent digestibility				
DMD	75.6	76.1	0.68	0.67
CPD	79.1	79.1	0.48	0.98
NDFD	49.4	49.9	1.48	0.86
ADFD	34.3	29.2	2.11	0.38

SQ, *Sasa quelpaertensis* Nakai; TMR, total mixed ration; SEM, standard error of mean; DMD, dry matter digestibility; CPD, crude protein digestibility; NDFD, neutral detergent fiber digestibility; ADFD, acid detergent fiber digestibility.
